# Supplementary figures and images for: Chromosomal neighbourhoods allow identification of organ specific changes in gene expression
Source: PLoS Comput Biol. 2021 Sep 10;17(9):e1008947. doi: 10.1371/journal.pcbi.1008947 (PMC8457456; doi:10.1371/journal.pcbi.1008947)

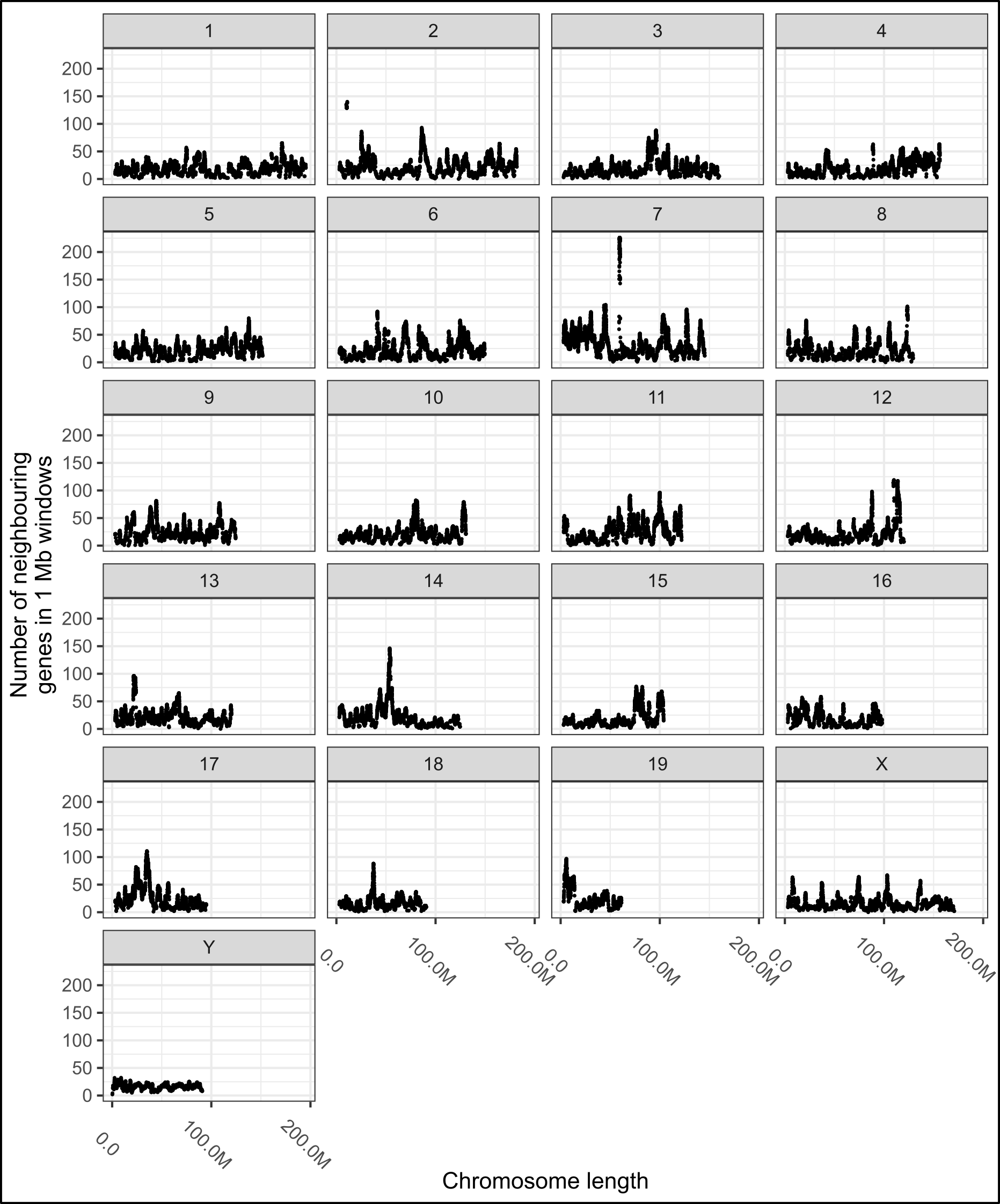

Supplement: S1 Fig — Distribution of genes across mouse chromosomes (x-axis) and number of “neighbouring genes” (y-axis) within 1 Mb window around each gene. (TIF) [file pcbi.1008947.s001.tif]

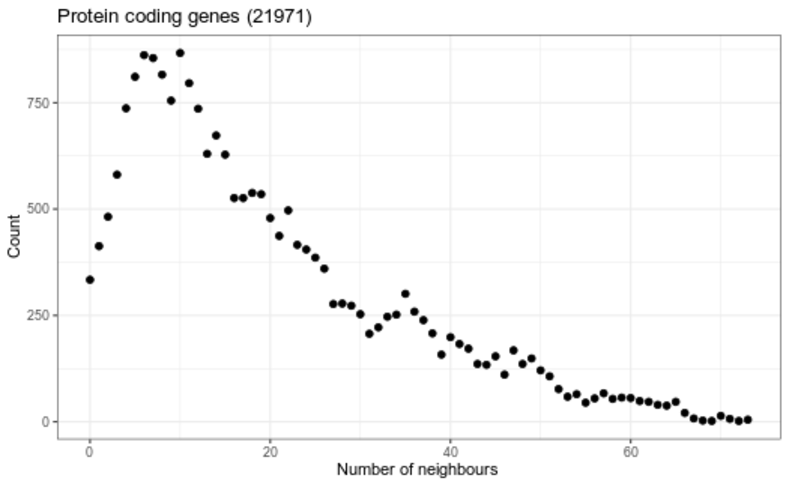

Supplement: S2 Fig — Each point represents the number of genes with the same number of neighbouring protein coding genes in 1 Mb window. (TIF) [file pcbi.1008947.s002.tif]

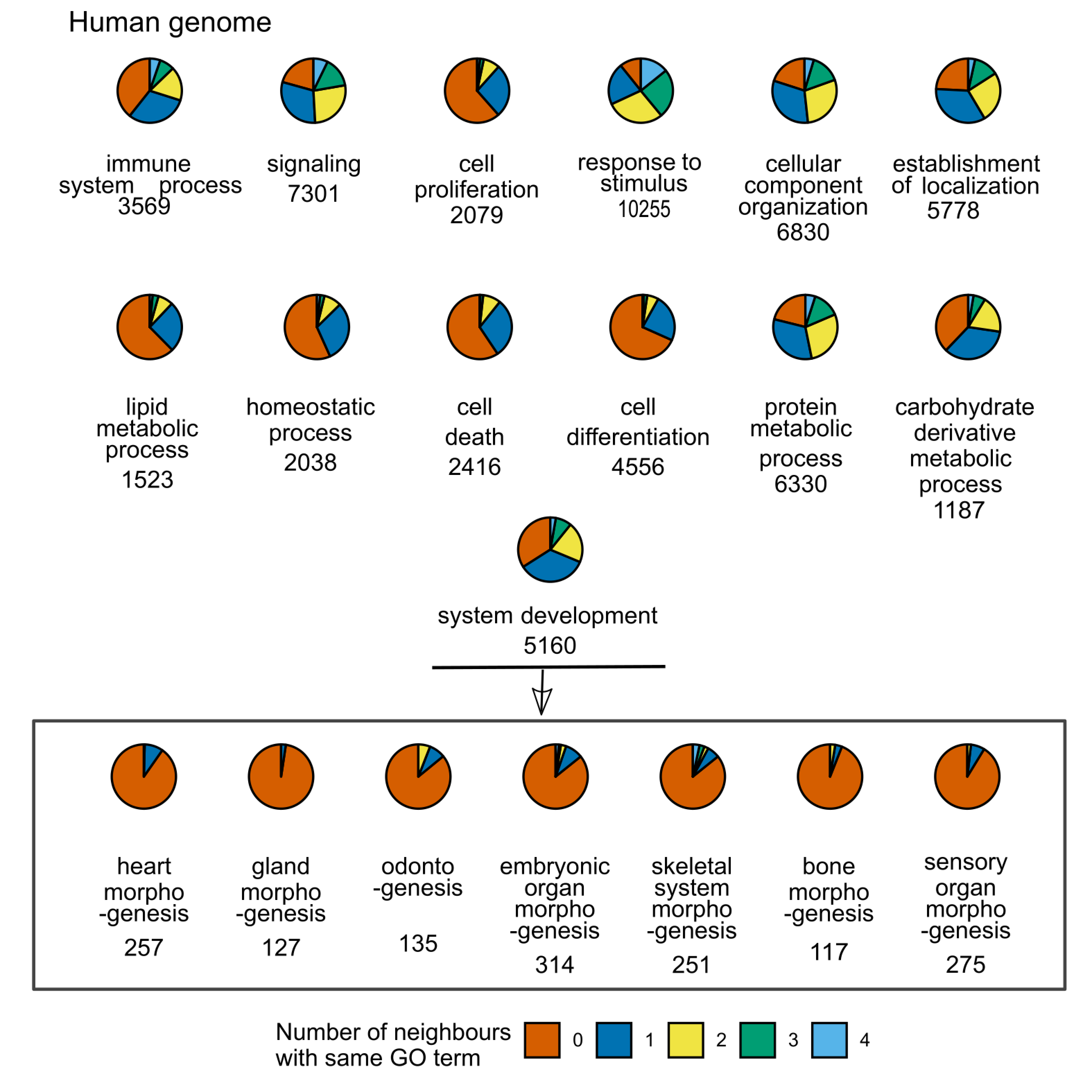

Supplement: S3 Fig — Each pie represents the genes of a GO term under the root GO term ‘biological process’. The GO terms are arranged from top to bottom following their proximity to the root term ‘biological process’. The color coding indicates the number of neighbours that a gene has from the same GO term. Analysis was done for the human genome. See S2 Table for GO IDs. (TIF) [file pcbi.1008947.s003.tif]

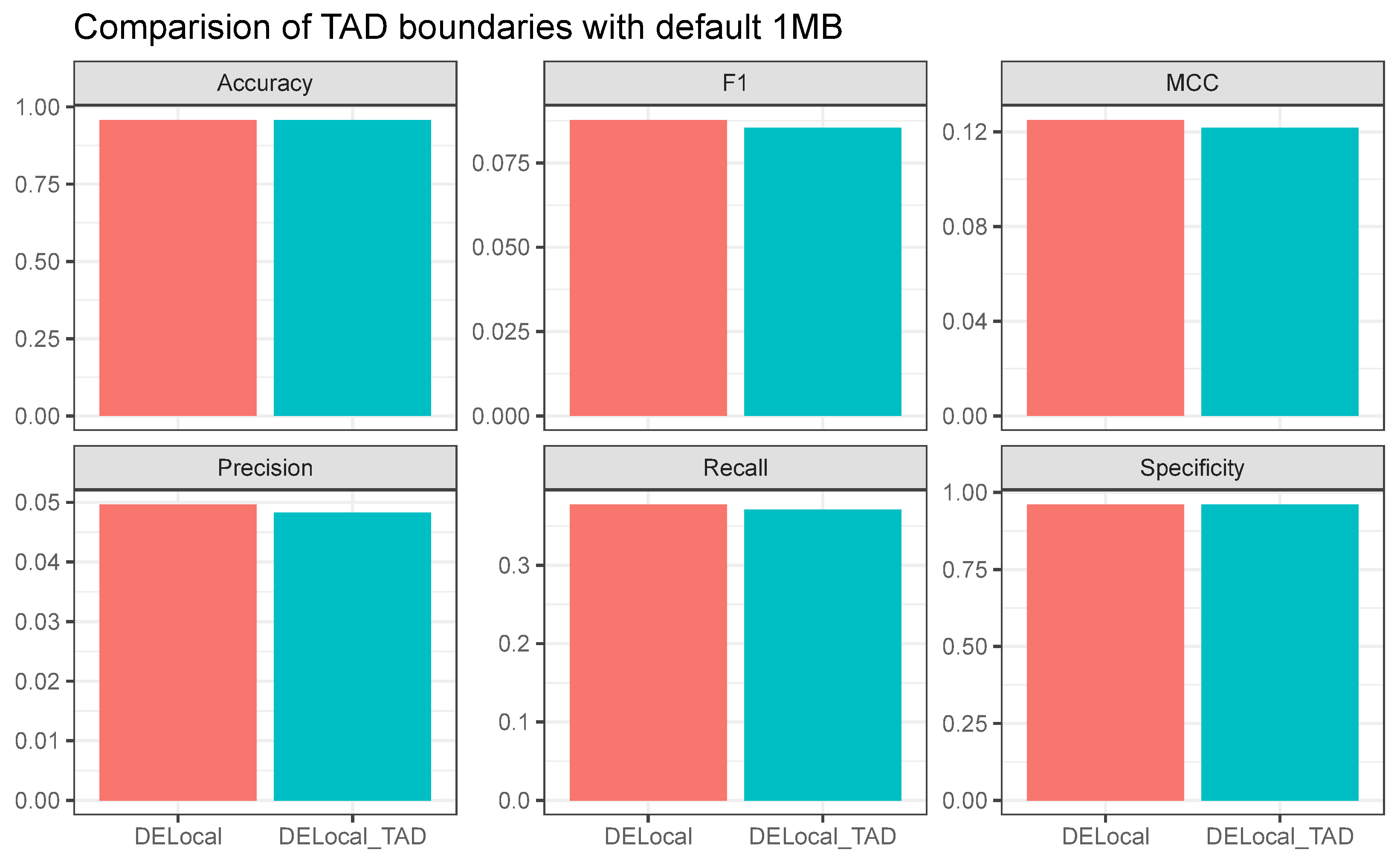

Supplement: S4 Fig — (TIF) [file pcbi.1008947.s004.tif]

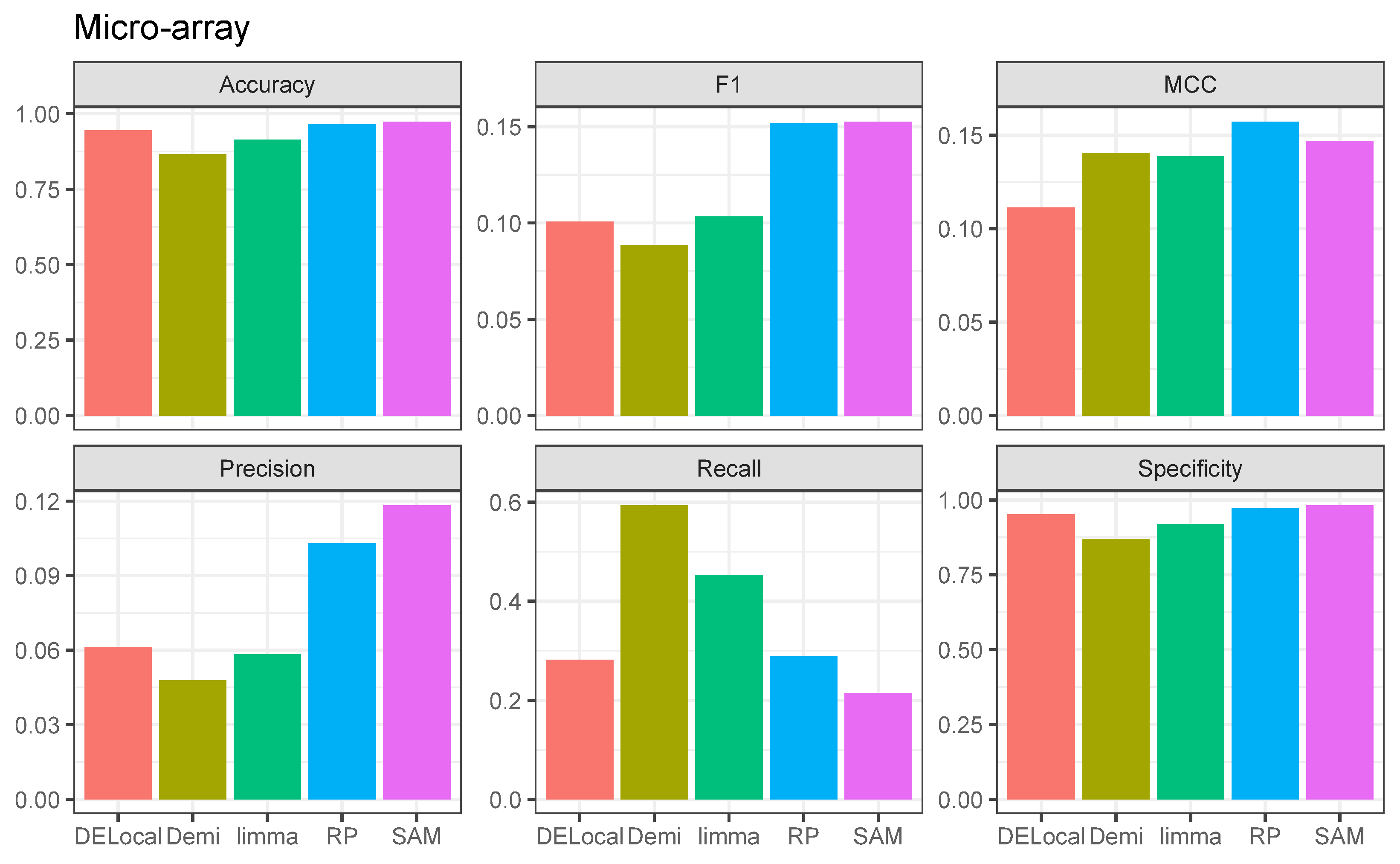

Supplement: S5 Fig — Evaluation matrices for microarray data. DELocal has lower performance in microarray data compared to RNAseq data (Fig 7), likely to be due to limited number of genes and neighbourhood data in microarrays. The evaluation matrices are explained in Materials and Methods section. (TIF) [file pcbi.1008947.s005.tif]

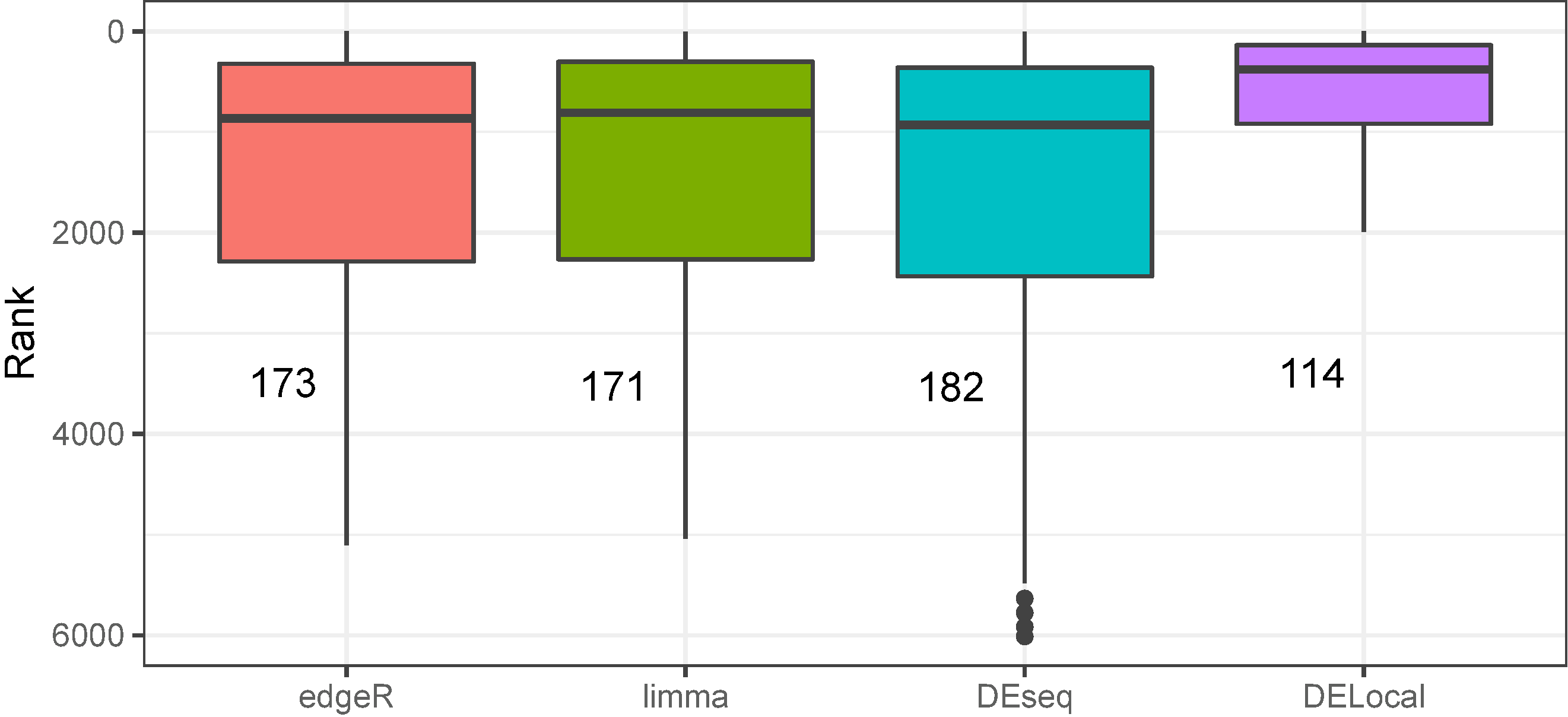

Supplement: S6 Fig — DELocal predicted tooth developmental genes are significantly (Wilcoxon rank test; p-value < = 1.0e-06) enriched in top ranked positions compared to the other three methods. Although other methods identified more of the genes (numbers listed next to the box plots), this improved recall is sacrificing specificity. DELocal balances both which is also reflected in F1, MCC and ROC curves (Figs 6 and 7). (TIF) [file pcbi.1008947.s006.tif]
