# Supplementary material for: Chromosomal neighbourhoods allow identification of organ specific changes in gene expression
Source: PLoS Comput Biol. 2021 Sep 10;17(9):e1008947. doi: 10.1371/journal.pcbi.1008947 (PMC8457456; doi:10.1371/journal.pcbi.1008947)
Supplement: S2 Table — (DOCX) [file pcbi.1008947.s008.docx]

Chromosomal neighbourhoods having genes of diverse functions allow improved identification of differentially expressed genes

Rishi Das Roy, Outi Hallikas, Mona M. Christensen, Elodie Renvoisé, Jukka Jernvall

**Table S2.** List of GO terms analysed in Figure 7,8 and S4.

| Display ID | GO ID | Description |
| --- | --- | --- |
| 1 | GO:0060349 | bone morphogenesis |
| 2 | GO:0042476 | odontogenesis |
| 3 | GO:0022612 | gland morphogenesis |
| 4 | GO:0048705 | skeletal system morphogenesis |
| 5 | GO:0003007 | heart morphogenesis |
| 6 | GO:0090596 | sensory organ morphogenesis |
| 7 | GO:0048562 | embryonic organ morphogenesis |
| 8 | GO:1901135 | carbohydrate derivative metabolic process |
| 9 | GO:0006629 | lipid metabolic process |
| 10 | GO:0042592 | homeostatic process |
| 11 | GO:0008283 | cell proliferation |
| 12 | GO:0008219 | cell death |
| 13 | GO:0002376 | immune system process |
| 14 | GO:0030154 | cell differentiation |
| 15 | GO:0051234 | establishment of localization |
| 16 | GO:0048731 | system development |
| 17 | GO:0019538 | protein metabolic process |
| 18 | GO:0016043 | cellular component organization |
| 19 | GO:0023052 | signaling |
| 20 | GO:0050896 | response to stimulus |
