# Supplementary material for: Chromosomal neighbourhoods allow identification of organ specific changes in gene expression
Source: PLoS Comput Biol. 2021 Sep 10;17(9):e1008947. doi: 10.1371/journal.pcbi.1008947 (PMC8457456; doi:10.1371/journal.pcbi.1008947)
Supplement: Attachment — Submitted filename: DeLocal_response.docx [file pcbi.1008947.s009.docx]

Evo-Devo lab

Institute of Biotechnology
P.O. Box 56 (Viikinkaari 9)
FIN-00014 University of Helsinki, FINLAND

Dear Editors,

Thank you very much for the reviews and the offer to consider a revised version of our manuscript. We found the referee comments very helpful in revising the paper as they pointed out aspects of the work we had overlooked. We feel these revisions have improved the manuscript substantially, especially as now the significance and novelty of the new method is explained for a broader readership. All the changes addressing referee comments have been highlighted. Additionally, we have revised the manuscript, figures and captions to follow the PLOS Computational Biology format, and improved the language.

**Reviewer 1 wished us to expand the discussion about genome organization, and to explain more clearly what the DELocal method offers for developmental biologists. We have highlighted the changes in the manuscript in blue color.**

Reviewer #1:

1) This manuscript first assesses the distribution of genes related to specific biological functions and organs. The functions were defined by gene ontology terms or experimental data from tooth development studies. It was found that even though most genes have several neighbors within 1 Mb, the neighboring genes rarely participate in the same function as might be expected if the bacterial operon control mode were followed in these systems.

It is well-known, however, that gene regulation in animals occurs by entirely different mechanisms than in bacteria, using expression hubs that bring together for coordinate expression linearly distant genes. So, although the results provided in this part of the paper are significant, they are not unexpected. One 18-year-old paper (ref. 19) that hints at this explanation is cited on p. 6, but it is not enough to dispel the impression that the result is surprising.

The authors have an excellent statement relating to this in their Discussion: “It remains to be explored whether the rarity of neighbouring genes regulating the same process…folding of the DNA, or whether it is the result of historical processes of genome rearrangements or co-option of genes in the evolution of organs.” But these ideas should also be raised in the Introduction in my opinion, and more recent treatments such as <https://pubmed.ncbi.nlm.nih.gov/30262496/> or <https://pubmed.ncbi.nlm.nih.gov/31226276/> should be cited.
**RESPONSE: We have added these references and expanded the introduction (lines 62-68, 82-92 and elsewhere (line 153) as requested. We make it hopefully more clear that the scattered distribution of organ specific genes fits well what is known, and also now discuss the topologically associating domains (TADs) as suggested by referee 2. Still, for developmental biologists, the lack of neighbouring genes regulating the same organs should be a worthwhile result, as also the DELocal method that is now explained much more clearly (see the next comment below).**

2) The paper then advances a new method, DELocal, to determine the relative activity of genes in chromosomal regions within a 1 Mb window in animal cells. Expression of genes during tooth development is used as an index case of coordinate expression in organogenesis.

The authors compare the performance of their DELocal method on microarray and RNASeq data (the latter being more abundant) to other methods for evaluating differential gene expression: SAM, DEMI, Limma, edgeR and DESeq2. The measures used were Sensitivity, Specificity, Precision, Accuracy, F1 score, and Mathews correlation coefficient. While these terms of art in the gene expression field are defined by algebraic expressions in the Methods section, they seem important enough for them to also be better described in the main text for the benefit of nonspecialist developmental biologists and others who would be interested in the qualitative implications of the paper.

**RESPONSE: The referee is right that we did not spell out the significance of DELocal, and we have now expanded the description of the results (also in response to referee 2 comment). Most notably, we have added a new section where we explain the significance of the DELocal to developmental biologists (lines 325-348). Specifically, by examining changes in gene expression by taking into consideration changes in the neighbouring genes, we can detect even subtle developmental changes in gene expression. What this means in practice is that DELocal removes the bias present in other methods to emphasize genes showing large changes in their expression. We have illustrated this in a new Figure 8 in which the rank order of differentially expressed genes is plotted against their log-fold change (Figure 8A). This is what developmental biologists first (and sometimes only) examine in their results. DELocal provides a substantially different list of top ranked genes compared to the other commonly used methods (that provide relatively comparable listings to each other, Figure 8B and Supplementary Fig. S6). We do not state that DELocal is necessarily ‘better’. Rather, depending which biological point of view one wants to explore, one can use DELocal as a ‘second opinion’ to analyze changes in gene activity during development. In addition to the figures and a new section, we now spell out this aspect of the method in abstract and discussion (lines 34, 47, 397-405).**

3) The result reported on pp. 12-13 that “DELocal missed 60 differentially expressed TDGs which are identified by all the other methods in [the] RNAseq dataset” is reasonably explained by the authors based on the assumptions of the method. But one can ask if the assumption-defying presence of unrelated differentially expressed genes in the vicinity of TDGs is related to peculiarities of the tooth system. Unlike, for example, cartilage or cardiac muscle, which have (relatively) specific lineage-determining transcription factors (Sox9 and Nkx3-1, respectively), and cell-type-specific products (type II collagen, cardiac myosin HC), tooth development seems to mobilize more generalized regulatory and function-mediating proteins, likely to reside in functionally general chromosome regions. If so, the tooth might represent a stringent challenge that establishes the rigor of the DELocal method which should actually work better for organogenesis involving greater specificity in gene expression.
**RESPONSE: This is an interesting possibility. All epithelial organs (e.g., teeth, hair, glands) have fairly similar early development and share largely the same expression profiles. So DELocal might be expected to perform similar among these organs, but these will indeed require further studies. We now mention the need to test other organs (lines 423-425).**

**In addition, we have improved the language as requested.**

**Reviewer 2 wished us to address the definition of the chromosomal in the context of topological association domains, and the statistical improvement of the method in comparison to existing methods. We have highlighted the changes in the manuscript in blue color.**

Reviewer #2: In this manuscript Das Roy et al., explore whether: 1) The "chromosomal neighborhood" of genes leads to correlated gene expression levels/patterns during organ development; and 2) If so, does accounting for these "chromosomal neighborhoods" improve estimates of (differential) gene expression. I find 1 to be an interesting question and 2 to be important if true. Unfortunately the methods and results do not currently support the conclusions of the authors that both 1 and 2 are true. I see two major concerns that undermine the authors conclusions:

1) The definition of "chromosomal neighborhood" is arbitrary and based on an a priori defined distances. Why should it be that 1Mb is the "right" size of a neighborhood and why should that size be the same for all chromosomal regions? This a priori definition is particularly problematic because there is a biological definition of a neighborhood: Topologically associating domains (TADs) and the smaller compartments (A and B). Previous studies have shown that both of these biologically defined neighborhood vary in size (unlike the 1Mb definition), location (cannot be tiled by 1Mb regions across the genome), and that genes within both TADs and A/B compartments have more or less similar expression levels. Thus, the arbitrary 1Mb regions is problematic. To overcome this limitation the authors should repeat the analyses using regions defined by TADs and A/B compartments from chromosome conformation studies (which might or not might exist for these tissues, but both tend to be conserved across tissues so could be used from other tissues) and/or repeat the analyses using variable distance tiles across the genome.

**RESPONSE: We chose the 1Mb search window as a straightforward approach that is readily implementable to diverse genomes. Note that DELocal uses expression levels of up to five nearest neighbours, thus the neighbouring region included in the pipeline is variable and typically less than 1Mb, a point we now emphasize as it was not stated clearly (lines 268-273). However, we fully agree with the referee that TADs and other compartments would be biologically more appropriate criteria for neighbourhood and we now looked into this in detail. Reviewing the recent literature was a bit surprising to us considering all the developments in the field. Among other things, associations of TADs with gene expression seem to be complex. Nevertheless, we now run the analyses using estimated TADs for the mouse, which did not however, alter the pattern of results. This is likely to be due to the mouse TADs to be generally in the vicinity of 1Mb in size. Regardless, as the topological association domains are dependent on the used algorithms and/or availability of Hi-C data, we prefer to keep the 1Mb pipeline in the paper as it provides the simplest way to extend the approach to other systems. We now discuss topological association domains in the introduction (lines 82-92, 103-108), methods (lines 454-465), results (lines 268-273 and supplementary figure S4), and discussion (lines 423-425). It is clear that as research on topological association domains progresses, methods such as DELocal can benefit from, as also help to contribute to, the progress.**

2) While accounting for 1Mb "chromosomal neighborhood" improves DE results, the effect size is very small from the ROC curves (0.02-0.03) and the data shown in figure 7. But, there is no data presented that these improvements are statistically significant. Please add analyses to demonstrate that these small effect sizes are statistically significant; perhaps bootstrapping the neighborhoods to generate confidence intervals and standard deviations.

**RESPONSE: The ROC curves provide visualization of the true positive and negative rates of discrimination, and do not indeed provide significance values by themselves. Yet, these are often used to guide the choice of methods and threshold of detection apart from significance tests (and DELocal appears to perform well in this respect). Now, this comment by the referee two and comment 2 by the referee one made us realize that we had managed to almost completely not communicate the most significant aspect of the DELocal. That is, by examining changes in gene expression by also taking into consideration changes in the neighbouring genes, we can detect even subtle developmental changes in gene expression. What this means in practice is that DELocal removes the bias present in other methods to emphasize genes showing large changes in their expression. We have illustrated this in a new Figure 8 in which the rank order of differentially expressed genes is plotted against their log-fold change (Figure 8A). This is typically the point where experimentalists are first viewing their results, and here DELocal provides substantially (and qualitatively) different gene ranking from the other methods. Indeed, the list of top ranked genes by DELocal is different from all the other commonly used methods that provide relatively comparable listings to each other (Figure 8B and Supplementary Fig. S6 for the statistical test). We do not state that DELocal is necessarily ‘better’. Rather, depending which biological point of view one wants to explore, one can use DELocal as a ‘second opinion’ to analyze changes in gene activity during development. In addition to the figures and a new section (lines 325-348), we now spell out this aspect of the method in abstract, discussion and method (lines 34, 47, 397-405, 546-553).**

The manuscript and its conclusions would be significantly improved should these concerns be addressed and the conclusions still warranted.

**RESPONSE: We feel the discussion and analyses using TADs (although preliminary) and the clarification on what the DELocal method really does should make the work much more generally interesting, and increase the likelihood somebody trying the DELocal.**

**In addition, we have made sure the empirical data is openly available (at GEO, accession number listed in the paper) and the DELocal scripts are available at** [**https://github.com/dasroy/DELocal**](https://github.com/dasroy/DELocal) **(see “Data Availibility” in manuscript).**

We thank again the referees for their comments and look forward to hearing from you.

Sincerely

Dr Rishi Das Roy and co-authors
